# 1683. Severe Group A *Streptococcu*s Infections Amongst Pediatric Patients in the Lehigh Valley, Pennsylvania Following the COVID-19 Pandemic

**DOI:** 10.1093/ofid/ofad500.1516

**Published:** 2023-11-27

**Authors:** Stormie Gough, Jonathan Berback, Tibisay Villalobos

**Affiliations:** Lehigh Valley Reilly Childrens Hospital, Allentown, Pennsylvania; Lehigh Valley Reilly Childrens Hospital, Allentown, Pennsylvania; Lehigh Valley Reilly Childrens Hospital, Allentown, Pennsylvania

## Abstract

**Background:**

In December 2022 the CDC issued an alert about possible increase of invasive group A *Streptococcus* infections (iGAS) among children in the United States. Colorado and Minnesota observed an increase in the number of cases in the Fall 2022. Similarly, the Pennsylvania Department of Health issued a health alert that was then lifted in February 2023. Preliminary CDC data showed that iGAS infections were higher in some areas of the country compared to pre-pandemic levels. Lehigh Valley Reilly Children’s Hospital is a community teaching hospital in Allentown, Pennsylvania. A rise in the number of children admitted with GAS infections was noted in the same period compared to previous years. The aim of our study was to determine the clinical characteristics of patients admitted with iGAS and non-iGAS infections during fall and winter 2022-2023.

**Methods:**

Retrospective chart review of patients 18 years and younger admitted to Reilly Children’s Hospital between September 1^st^ 2022 through March 31^st^ 2023 and diagnosed with GAS infection plus those admitted with the same diagnosis between 2018 and 2022.

**Results:**

There were 19 children admitted to the hospital with GAS infection: 6 (32%) with iGAS and 13 (68 %) with non-iGAS infections. The iGAS infections included bacteremia without source (2), myositis (1), pneumonia (2), and vascular infection (1). Non-iGAS infections included retropharyngeal abscess (3), peritonsillar abscess (2), parotid abscess (1), submandibular abscess (1), lymphadenitis (2), mastoiditis (1), cellulitis (1), pharyngitis (1), and erythema nodosum (1). Six children required intensive care and two were transferred to higher level care. Median age was 2.2 year for iGAS infections and 4.8 years for non-iGAS. All but one patient had no underlying medical conditions. There were no patient deaths. In 2018 there were zero cases of iGAS infection; in 2019: two cases; 2020: two cases; 2021: zero cases. There were no admissions between May 2020 and April 2022 for either iGAS or non-iGAS.

**Conclusion:**

The number of children admitted for iGAS and non-iGAS infections in the fall and winter of 2022-2023 surpassed the preceding 4 years combined. This is reflective of what was happening in some other areas of the country as a result of reduced exposure and lack of immunity due to pandemic restrictions.

**Disclosures:**

**All Authors**: No reported disclosures

